# Impedance planimetry-guided peroral endoscopic myotomy of the fundoplication valve

**DOI:** 10.1055/a-2291-9572

**Published:** 2024-04-09

**Authors:** Sandra Nagl, Alanna Ebigbo, Marc Barthet, Helmut Messmann

**Affiliations:** 1 39694 Department of Gastroenterology, University Hospital Augsburg, Augsburg, Germany; 2 Department of Hepatogastroenterology, Assistance Publique des Hôpitaux de Marseille Aix-Marseille, Université Hôpital Nord Marseille, Marseille, France

We present a novel approach involving impedance planimetry with the endoscopic functional lumen imaging probe (EndoFLIP) to guide peroral endoscopic myotomy of the fundoplication valve (FP-POEM) ( Video 1 ).

Endoscopic myotomy of the fundoplication valve is performed with intraoperative guidance via impedance planimetry in a patient with dysphagia following laparoscopic fundoplication surgery.Video 1

A 34-year-old man presented with severe dysphagia following three laparoscopic fundoplications and revisional surgical interventions for gastroesophageal reflux disease (GERD). He was unable to consume solid foods, necessitating parenteral feeding. Radiologic and endoscopic evaluations were similar to type I achalasia, characterized by esophagogastric junction (EGJ) tightening and a dilatation of the distal tubular esophagus. Previous surgical interventions had resulted in pronounced scarring and fibrosis, making further surgical interventions to release the fundoplication unfeasible. After a multidisciplinary review, we opted for a third-space endoscopic approach to dissect the fundoplication valve 1 .

Prior to and after the myotomy, intraoperative impedance planimetry with EndoFLIP was used to assess distensibility, yielding a distensibility index (DI) of 1.7 and 1.5 mm ^2^ /mmHg with 30-mL and 40-mL balloons, respectively, prior to myotomy. A posterior tunnel was initiated 5 cm proximal to the EGJ and extended 3 cm into the fundoplication site. The myotomy involved the circular esophageal muscles and the fundoplication valve. Owing to the pronounced fibrosis, the exact extent of the myotomy was difficult to predict correctly. Post-myotomy, the DI improved to 2.8 mm ^2^ /mmHg. With intraoperative guidance from EndoFLIP in standard esophageal POEM for achalasia, the objective is to enhance the DI by a minimum of 200% 2 . Consequently, further myotomy was performed, resulting in a DI of 5.1 and 4.5 mm ^2^ /mmHg with the 30- and 40-mL balloons, respectively. The tunnel entrance was secured with four hemoclips.

At the early follow-up assessment after 3 months, the patient’s symptoms had resolved. He was able to eat solid foods and had no complaints of reflux.

This represents the first case of EndoFLIP-guided FP-POEM. The use of intraoperative DI and cross-sectional area evaluations allowed optimization of the myotomy extent. While EndoFLIP has been used in standard esophageal POEM 3 , its potential utility may be particularly pronounced in more challenging cases.

Endoscopy_UCTN_Code_CPL_1AH_2AH

## Notes

Endoscopy E-Videos https://eref.thieme.de/e-videos
*E-Videos* is an open access online section of the journal *Endoscopy* , reporting on interesting cases and new techniques in gastroenterological endoscopy. All papers include a high-quality video and are published with a Creative Commons CC-BY license. Endoscopy E-Videos qualify for HINARI discounts and waivers and eligibility is automatically checked during the submission process. We grant 100% waivers to articles whose corresponding authors are based in Group A countries and 50% waivers to those who are based in Group B countries as classified by Research4Life (see: https://www.research4life.org/access/eligibility/ ). This section has its own submission website at https://mc.manuscriptcentral.com/e-videos .
